# Bivariate Joint Spatial Modeling to Identify Shared Risk Patterns of Hypertension and Diabetes in South Africa: Evidence from WHO SAGE South Africa Wave 2

**DOI:** 10.3390/ijerph18010359

**Published:** 2021-01-05

**Authors:** Glory Chidumwa, Innocent Maposa, Paul Kowal, Lisa K. Micklesfield, Lisa J. Ware

**Affiliations:** 1 Division of Epidemiology and Biostatistics, School of Public Health, University of the Witwatersrand, Johannesburg 2198, South Africa; innocent.maposa@wits.ac.za; 2 World Health Organization SAGE, CH-1211 Geneva, Switzerland; paul.r.kowal@gmail.com; 3 Research Institute for Health Sciences, Chiang Mai University, Chiang Mai 50200, Thailand; 4 South African Medical Research Council/Wits Developmental Pathways for Health Research Unit, School of Clinical Medicine, University of the Witwatersrand, Johannesburg 2198, South Africa; Lisa.Micklesfield@wits.ac.za (L.K.M.); lisa.ware@wits.ac.za (L.J.W.); 5 DSI-NRF Centre of Excellence in Human Development, University of the Witwatersrand, Johannesburg 2198, South Africa

**Keywords:** hypertension, diabetes, joint shared spatial model

## Abstract

Recent studies have suggested the common co-occurrence of hypertension and diabetes in South Africa. Given that hypertension and diabetes are known to share common socio-demographic, anthropometric and lifestyle risk factors, the aim of this study was to jointly model the shared and disease-specific geographical variation of hypertension and diabetes. The current analysis used the Study on Global Ageing and Adult Health (SAGE) South Africa Wave 2 (2014/15) data collected from 2761 participants. Of the 2761 adults (median age = 56 years), 641 (23.2%) had high blood pressure on measurement and 338 (12.3%) reported being diagnosed with diabetes. The shared component has distinct spatial patterns with higher values of odds in the eastern districts of Kwa-Zulu Natal and central Gauteng province. The shared component may represent unmeasured health behavior characteristics or the social determinants of health in our population. Our study further showed how a shared component (latent and unmeasured health behavior characteristics or the social determinants of health) is distributed across South Africa among the older adult population. Further research using similar shared joint models may focus on extending these models for multiple diseases with ecological factors and also incorporating sampling weights in the spatial analyses.

## 1. Introduction

The global burden due to non-communicable diseases (NCDs) is high and rising; and is expected to increase in the next decades if public health interventions are not implemented to reduce the trend [1,2,3]. In sub-Saharan Africa, the total number of disability-adjusted life years (DALYs) due to NCDs for all ages increased by 67% between 1990 and 2017 [4]. Among African countries, this epidemiological transition has been reported to be attributable to the changing lifestyle patterns such as declining levels of physical activity [5,6]. In South Africa, NCDs have become the leading cause of mortality accounting for 40% of total deaths, with one-third of the deaths occurring before the age of 60 [7]. Recent studies have further highlighted the co-occurrence of NCDs. Oni and colleagues have highlighted the co-existence of multiple infectious diseases and NCDs in Cape Town adults from an informal settlement [8]. Their findings showed a 23% prevalence of multimorbidity (defined as having more than one chronic condition) among chronic disease patients, and patterns of multimorbidity with hypertension and diabetes often co-occurring [8]. In addition, in South Africa, non-diabetics with elevated blood pressure are 2.5 times more likely to develop diabetes within 5 years than individuals with normal blood pressure levels [9]. Individuals living with diabetes are twice as likely to have hypertension [10].

Data across multiple conditions may be pooled in a unified way using joint mapping models to better understand the overlapping epidemiology of the conditions. The present study used a bivariate spatial disease model to analyze hypertension and diabetes simultaneously [11,12,13]. Such multivariate models have been used for the following reasons, Firstly, the correlation structures between relative risks of related diseases is implicitly quantified. Secondly, the models enable common and disease-specific observed covariate effects as well as spatial patterns at the same time [12,13]. Similar work has used joint mapping models in cancer research, childhood illnesses, and childhood cancer research as well as diabetes research [14,15,16].

The current study aims to assess the shared component risk profile for hypertension and diabetes using data from the World Health Organization (WHO) Study on Global Ageing and Adult Health (SAGE) South Africa Wave 2. These conditions are known to share common socio-demographic, anthropometric and lifestyle risk factors [10,17]. Therefore, the shared component can be interpreted as a proxy for unobserved covariates that display spatial structure and are common to both diseases. Similarly, each disease-specific component represents spatially varying risk factors which are specific to the respective disease.

## 2. Materials and Methods

In this study, we used data from WHO SAGE South Africa Wave 2. SAGE is an ongoing multi-country longitudinal study that has also been implemented in China, Ghana, India, Mexico, and the Russian Federation. SAGE aims to examine the health and wellbeing of nationally representative adult populations aged 18+ years with an emphasis on populations aged 50+ years [18]. Further details are available at (http://www.who.int/healthinfo/sage/en/). In South Africa, 600 enumeration areas, with 30 households in each, were sampled from 18,000 targeted households. In the sample of households with people aged 50 years or older, all adults aged 50 years or older were eligible for interview. SAGE South Africa wave 1 total sample size for South Africa was 4223. Wave 2 is an implementation of the SAGE follow-up of Wave 1, 7 years later. In this sample, approximately 30% of participants interviewed in Wave 1 were interviewed again at Wave 2 and were linked using their unique identifiers. The remainder were new participants. The current analysis consists of 2761 participants who had valid (not equal to zero) post-stratification weights, with full data on hypertension and diabetes. The provincial samples of the participants who were 50 years and older in our study were representative of the middle-aged and older population in each province as shown in Supplementary Table S1.

**Figure 1:**
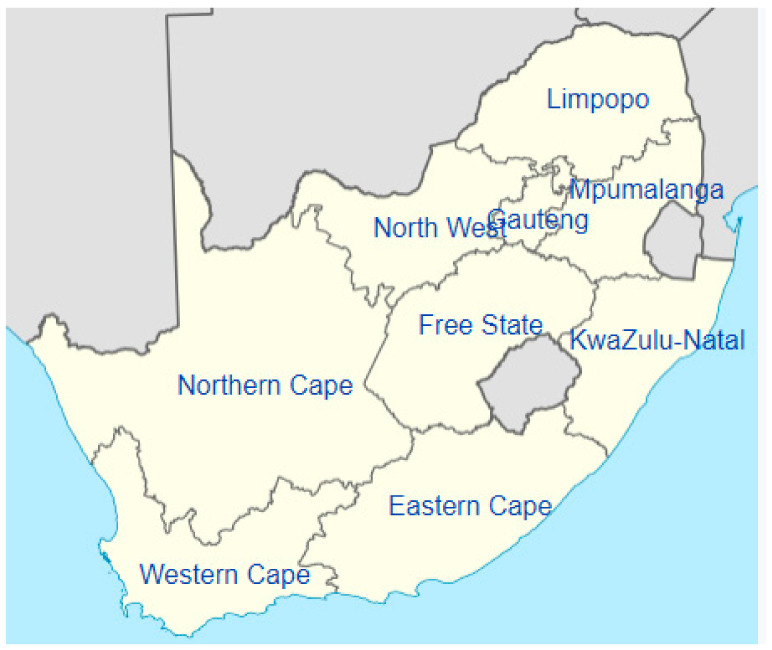
The South African map [Source: https://en.wikipedia.org/wiki/Provinces_of_South_Africa].

The unit of analysis for the current spatial model is district. The SAGE study used province and residence as the main stratification levels. South Africa consists of three structures of government—national, provincial and local governments—and is divided into nine provinces, each with a provincial legislature (see Figure 1). The nine provinces are further divided into 52 districts. Provincial governments are bound by laws and policies passed at national level. However, provincial governments can adapt or develop their own laws and policies within the national framework to suit their specific needs [19]. The National Health Act requires provincial Departments of Health to develop their own strategic plans, which must conform with national health policy [20]. The provincial governments, therefore, implement their own priorities and allocate resources responsive to the needs of their populations.

Gauteng province is the most densely populated province with nearly eight hundred people per square kilometre, followed by Kwa-Zulu Natal province (178 people per square kilometre). The least densely populated province is the Northern Cape, with on three people per square kilometre. Gauteng contributes to approximately a third (34%) of South Africa’s growth domestic product. The Eastern Cape and Limpopo provinces have the highest percentages of households in poverty, 12.7% and 11.5%, respectively, while Western Cape has only 2.7% households in poverty.

Outcome variables: Hypertension status was determined as a measured average (for three sequential readings) systolic blood pressure (SBP) readings of ≥140 mmHg and/or an average diastolic blood pressure (DBP) reading of ≥90 mmHg and/or self-reported hypertension with current use (within the last 2 weeks) of antihypertensive medication [10]. For the present descriptive analysis, participants with systolic and diastolic blood pressure (BP) values <120/80 mmHg were classified as normotensive, while those with systolic BP from 120 to 139 mm Hg and diastolic BP from 80 to 89 mm Hg as prehypertensive. Self-reported diabetes status was assessed with the question “Have you ever been told by a health professional/doctor that you have diabetes?”.

“Exposure and predictor variables: Demographic variables included age, sex, years of schooling completed, and area of residence (urban or rural). Behavioural and social variables included ever used alcohol, ever used tobacco (smoked and smokeless), adding salt at the table (yes/no), self-reported vigorous intensity physical activity (yes/no—both leisure and work), and current employment status [18]. Anthropometric measures included waist circumference, weight and height; and were measured in accordance with WHO standardised techniques with all fieldwork teams trained by WHO staff. Waist to height ratio [waist (cm)/height (cm)] and body mass index were calculated [weight (kg)/height (m)^2^). For descriptive purposes, we classified participants into the categories ‘underweight’ (body mass index (BMI) < 18.5 kg/m^2^), ‘normal weight’ (BMI > 18.5 and < 25.0 kg/m^2^), ‘overweight’ (BMI > 25 kg/m^2^) or ‘obese’ (BMI > 30 kg/m^2^). Details about the WHO standardised interview and direct measurement techniques are described elsewhere [18]. Principal components analysis (PCA) was used to derive a socioeconomic status (SES) index for each household. PCA involved using household ownership of a set of 19 assets, household density and household service access (sanitation and electricity) into categorical or interval variables. The variables were then processed in order to obtain weights and principal components. The results obtained from the first principal component (explaining the most variability) were used to develop an index. The SES indices were then grouped into household wealth tertiles, reflecting different SES levels in the wealth continuum, as previously applied [21,22]. Financial support from government (yes/no) was also determined.

### Statistical Methods

For the analyses, we used the following versions of software and packages: Stata Release 16.1 (Stata Corp LLC., 2017; College Station, TX, USA) and OpenBUGS version 3.2.3 rev 1012 [23]. Individual level data summary statistics, and provincial level prevalence of hypertension and diabetes were determined. In order to counter any possible negative confounding, individual factors to include in the final spatial shared models were selected a priori and using stepwise backward elimination multiple logistic regression for hypertension and diabetes separately (*p* = 0.1). The current analysis incorporates an ecological investigation to assess disease risk in relation to risk at individual level exposure. Joint disease mapping models are a direct extension of univariate spatial models that use both global and local spatial dependence structures to model risk of diseases. The extensions enable analysts to make an assessment on similarities as well as differences between risk factors for diseases which share common risk profiles [11,14,15,16,24,25]. One such joint disease mapping model is the shared-component model which fits common and disease-specific unobserved and unmeasured spatial risks [12,14,24]. A detailed description of the shared-component model and its implementation within the Bayesian estimation procedure for hypertension and diabetes is given below:

Each j district (*j* = 1, …, 52) has $N_{j}$ adults out of the total sampled, i.e., the sample of adults in the study from the total district population. We assume that $X_{ij}$ is the vector of observed risk covariates associated with a subject i (*i* = 1, …, 2761) in district j. For the unaccounted variation in the risks of hypertension and diabetes, unobserved district-spatial variation $U_{jk}$ is introduced for district j and NCD k (*k* = 1 and 2). We worked within the framework of conditional models where conditional on spatial random effects $U=\;\left(U_{j1}\;and\;U_{j2}\right)$ and the disease-specific fixed effects parameters $\beta_{k}$, the binary responses $Y_{ijk}$ were independent Bernoulli random variables with parameters $\pi_{ijk}$, being the probability of subject ij having disease k. In order to model the probabilities of the observed and unobserved spatial variation, we used a logit link function on the probabilities (i.e., joint spatial model without the shared component):

(1) $$\log\left(\pi_{ijk}/\left(1-\pi_{ijk}\right)\right)=\alpha_{k}+\beta_{k}^{’}X_{ij}+U_{jk}+\varepsilon_{j}$$

where $\alpha_{k}’s$ are the disease-specific log-odds constant terms.

In order to model hypertension and diabetes together in a multivariate space, we used a shared-component model with one shared component, relevant to hypertension and diabetes. The shared spatial component could be interpreted as a proxy for variations in latent and unmeasured health behavior characteristics or the social determinants of health in our population. The known behavioral risk factors in our study include alcohol use, tobacco use, and adding salt at the table. We compared shared spatial component model with a spatial joint model to assess whether this model was better in capturing the underlying covariance structure of the data using the deviance information criteria (DIC). Within the symmetric formulations of the shared-component model, we also included disease-specific spatial components for hypertension and diabetes. Thus, the model decomposed each of the two spatial random effects $U_{j1}\;and\;U_{j2}$ into a common spatial and disease-specific component. The resulting model enables us to determine the extent of the variation exhibited through common as well as specific geographical patterns in the risks. We also allow for disease-specific unstructured heterogeneous effects $\epsilon_{jk}$ to account for possible extra-binomial variation that was not explained by the included fixed effect; and common and specific structured spatial terms. Thus, hypertension and diabetes were modelled as follows on log-odds scale (i.e., joint spatial model with the shared component), an extension of Equation (1):

(2) $$\log\left(\pi_{ij1}/\left(1-\pi_{ij1}\right)\right)\;=\alpha_{1}+\beta_{1}^{’}X_{ij}+\gamma_{1}Uj+U_{j1}+\varepsilon_{j1}$$

(3) $$\log\left(\pi_{ij2}/\left(1-\pi_{ij2}\right)\right)\;=\alpha_{2}+\beta_{2}^{’}X_{ij}+\gamma_{2}Uj+U_{j2}+\varepsilon_{j2}$$

where $U_{j1}\;and\;U_{j2}$ are the log-odds structured random effects for each of hypertension and diabetes, in district j. $\gamma_{i}$’s represent the risk gradient. The parameters $\alpha_{k}’s$ and $\beta_{k}^{’}$ ‘s are the disease-specific baseline risk and fixed effect risks associated with the risk vector $X_{ij}$; and $U_{j}$ is the shared component common to hypertension and diabetes.

## 3. Results

A total of 2761 adults provided the full set of health variables for our analysis, of which 641 (23.2%) had hypertension and 338 (12.3%) had diabetes. The median age was 56 years (inter-quartile range: 40–66 years). Mpumalanga province and Western Cape had the highest prevalence of hypertension (33.8% and 31.2%, respectively), while approximately one in five people are diabetic in Kwa-Zulu Natal and Western cape. Approximately 10% (n = 240) of our sample had comorbidity, defined as having both hypertension and diabetes. Comorbidity was significantly associated with demographic (age, sex and current employment), socioeconomic (years of schooling, high school completion, household wealth tertile, receiving government support), anthropometric (waist-to-height ratio, BMI), and behavioral characteristics (adding salt at table). A detailed summary of the individual-level data and bivariate analyses are shown in Table 1 and Table 2 below.

Results from the stepwise backward elimination multivariable analyses for hypertension and diabetes are shown in Supplementary Table S2. Increasing age was positively associated with increased risks of both hypertension and diabetes. Being unemployed and having a few years of education were associated with higher hypertension and diabetes risks, although employment was not statistically significant. In addition, higher waist-to-height ratio was positively associated with increased risks of both hypertension and diabetes. Participants who had self-reported diabetes and depression had 84% and 66% higher risk of hypertension relative to their controls, respectively. Table 3 below shows the multivariate logistic model, adjusting for demographic, socioeconomic status, anthropometry, behavioral characteristics and spatial effects. Age was positively associated with increased odds of both hypertension and diabetes. Being in the lower household wealth tertile and having fewer years of schooling were associated with higher hypertension and diabetes risks. In addition, receiving support from government, adding salt to food at the table and having a higher BMI were associated with greater odds of hypertension. Moreover, tobacco use was associated with increased odds of diabetes. Adjusted odds ratios and credible intervals are shown in Table 3 below.

**Figure 2:**
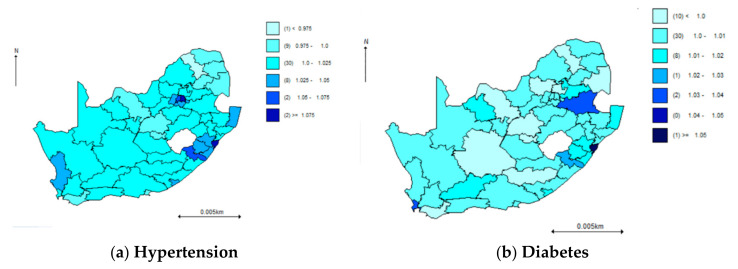
Odds of disease-specific components for (**a**) hypertension, and (**b**) diabetes, by district, World Health Organization (WHO) Study on Global Ageing and Adult Health (SAGE) South Africa Wave 2.

Figure 2 below shows the disease-specific spatial distribution of the covariate-adjusted estimated odds for hypertension and diabetes. The highest spatial distribution of the covariate-adjusted estimated odds for the disease specific component of hypertension was found in some parts of Kwa-Zulu Natal, Western Cape and Gauteng areas. For diabetes, risk was found to be highest in eastern provinces of Kwa-Zulu Natal, followed by Cape Town in the Western Cape, and Mpumalanga, with the lowest in Limpopo and Eastern Cape provinces. Of note, the odds of both disease-specific components and the shared component are high in more urbanized provinces compared to the poor provinces such as the Eastern Cape, as shown in the methods. The current shared component joint spatial model had better model fit relative to a joint spatial model without the shared component (DIC = 637.2 and 1.2 × 10^13^, respectively). This is in keeping with the comparison between Equation (1) versus Equations (2) and (3), which hypothesize that the joint shared model has a better model fit relative to the joint model without the shared model.

**Figure 3:**
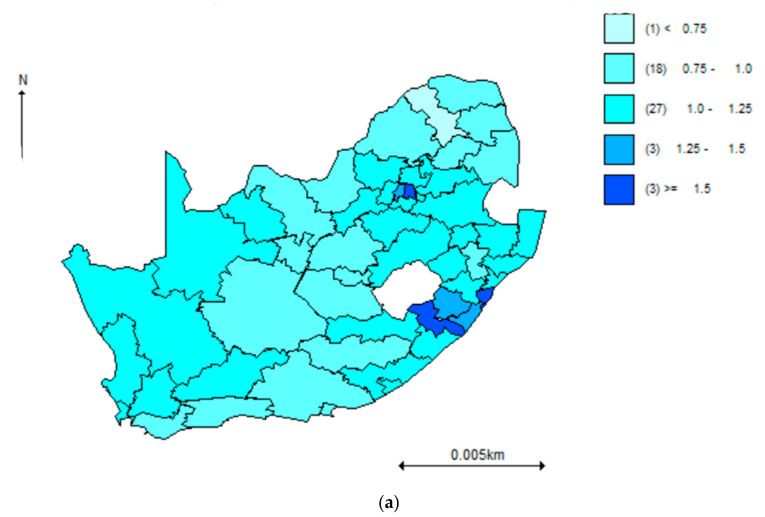

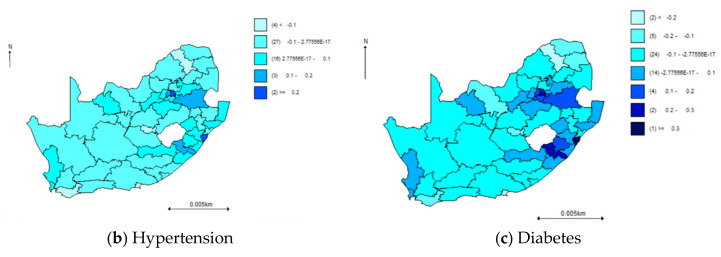
(**a**): Odds of the shared component in the joint (hypertension and diabetes) shared model, (**b**,**c**): Odds of the shared component risk contribution maps for hypertension and diabetes, by district, WHO SAGE South Africa Wave 2.

Figure 3a below displays the estimated shared component for the joint model. Of note, the shared component has distinct spatial patterns with higher values of odds in the eastern districts of Kwa-Zulu Natal and central Gauteng province. The fraction of total variation in odds ratios for hypertension and diabetes that is explained by the shared component were 0.67 and 0.53, respectively. The larger fractional contribution of hypertension to the shared component may explain the coincidental distribution between the shared estimates and hypertension estimates as shown on Figure 2 and Figure 3a. Figure 3b,c show the odds of the shared component risk contribution maps for hypertension and diabetes to indicate the absolute magnitude of shared odds for hypertension and diabetes. In addition, residual spatial effects may be more visible for diabetes than for hypertension given that the model may have explained more of hypertension.

## 4. Discussion

Our study used a shared joint spatial analysis to examine the spatial distribution of hypertension and diabetes and the potential role of latent and unmeasured socioeconomic status and health behavior characteristics or the social determinants of health (the shared component) on hypertension and diabetes in South Africa. Our results suggest that latent and unmeasured health behavior characteristics or the social determinants of health may have greater influence on hypertension and diabetes in the southern and central-eastern areas of the country. Common shared behavioural risks for hypertension and diabetes included in our analyses include physical activity, tobacco use, alcohol use, and salt use. The shared latent and unmeasured health behavior characteristics in our model may include ecological factors and environmental determinants such as population density, pollution, transport, power, and local food environment The shared component specifically indicates patterns of unobserved common effects and risk factors for hypertension and diabetes in the Western Cape, Gauteng, Kwa-Zulu Natal and Mpumalanga. The spatial distribution of the shared component of hypertension and diabetes in these areas is consistent with the distribution of established risk factors such as adoption of a sedentary lifestyle and urbanization in these regions [25]. The clusters also coincide with regions with high levels of socio-economic inequality [25]. In addition, the finding that the shared component indicated a greater influence on hypertension and diabetes in the Western Cape is consistent with previous literature. Joint disease mapping models have been previously used in the investigation of the distribution of cardiovascular conditions [11,12,13]. In a study by Kandala and colleagues aimed at estimating the spatial coexistence of coronary heart disease (CHD), hypertension, hypercholesterolaemia, and stroke among 13,827 South African adults from the South African Health and Demographic Survey found that the shared component, which they took to represent “nutrition and other lifestyle factors” not controlled for in their model, had a greater effect on cardiovascular disease prevalence in Western Cape and Northern Cape. This is despite the fact that their study was representative of the general adult population (18+ years) while our study is focused on adult population age 50+ years.

The high prevalence of diabetes is indicative of the social determinants of health showing that these cardiometabolic conditions disproportionately impact vulnerable members of our society in our setting as described in the methods. A population-level effort to reduce risks for hypertension (salt intake) was instituted in South Africa starting in 2016 with an aim to contribute to lowering high blood pressure across all provinces (Ware 2017) [26]. The finding in this study that older age, higher BMI, and being female were associated with hypertension was in agreement with previous literature in univariate analyses [10]. Unlike some other studies, this study did not find being physically inactive to be associated with hypertension and diabetes among the older adult population [27,28,29,30]. This could be due to the fact that physical activity was based on self-report rather than an objective indicator, and can thus be affected by possible recall bias and social desirability bias. Previous studies conducted in Europe and North America has showed that the prevalence of hypertension is consistently higher among men compared to women across different countries [31]. This could be attributed to the fact that in our population, healthcare utilization has been found to be associated with sex, with women being more likely to seek healthcare compared to men [32,33]. In addition, high BMI has been found to be associated with increased higher risk of cardiovascular diseases, particularly hypertension [34]. Obesity has also been reported to increase cardiovascular disease risk [10]. In addition, our findings that diabetes was associated with lower education and lower household wealth category were in keeping with previous literature [35]. This could be attributed to limited access to healthcare, and poor nutrition among participants in the lower wealth tertiles. Thus, this could suggest that the social determinants of health show that hypertension and diabetes disproportionately impact vulnerable members of society in the older South African population.

A key strength of SAGE is that it consists of nationally representative samples for the older adult population, with high response rates. The current study should, however, be viewed in light of the following limitations. Firstly, our models assume that the shared and specific components are independent, which ignores the possibility of interactions between the true covariates. Secondly, the analysed health data pose a possibility of under-reporting of diabetes in addition to the lack of objective measurements on habits such as tobacco use, alcohol use and physical activity. Furthermore, SAGE data had a larger representation of the Black African population and older adults (aged 50 years and older). We were not able to explore the influence of ethnicity on our models as the current sample only had 3% white participation rate as compared to the estimated 9% representation of the white population within South Africa. Nonetheless, the shared component model used in this study may be extended to the joint analysis of three or more diseases to understand unexplained common risk factors. Furthermore, joint modeling helps to stabilize parameter estimates in small area estimation where sample sizes at sub-regions with respect to each disease are small. In epidemiology, joint modeling may be useful in identifying similar patterns of disease and understanding diseases association.

## 5. Conclusions

The co-occurrence of hypertension and diabetes remains a concern in South Africa. Our study further showed how a shared component is distributed across South Africa among the older adult population. We further illustrate how this shared component is likely to influence the geographic distribution of hypertension, and diabetes in South Africa. Policy-makers may potentially use our spatial results for purposes of resource allocation and education in public health programs targeted to reduce the burden of hypertension and diabetes in South Africa, and also to manage this co-occurrence concurrently. In addition, further research using similar shared component joint models may focus on extending these models for multiple diseases with ecological factors and also incorporating of sampling weights in the spatial analyses.

## Figures and Tables

**Table 1:** Prevalence of hypertension, diabetes and comorbidity (having both hypertension and diabetes or not), by province (N = 2761).

PROVINCE	Total	Hypertension	Diabetes	Comorbidity
	N	N (%)	N (%)	N (%)
**Eastern Cape**	522	82 (15.7)	31 (5.9)	18 (3.5)
**Free State**	216	51 (23.6)	22 (10.2)	13 (6.0)
**Gauteng**	528	117 (22.2)	80 (15.2)	55 (10.4)
**Kwa-Zulu Natal**	450	127 (28.2)	86 (19.1)	65 (14.4)
**Mpumalanga**	142	48 (33.8)	14 (9.9)	13 (9.2)
**North West**	318	68 (21.4)	26 (8.2)	14 (4.4)
**Northern Cape**	93	19 (20.4)	10 (10.8)	7 (7.5)
**Northern Province**	175	30 (17.1)	8 (4.6)	6 (3.4)
**Western Cape**	317	99 (31.2)	61 (19.3)	49 (15.5)

All values are frequencies and percentages in parenthesis.

**Table 2:** Summary statistics for demographic, socioeconomic status, anthropometry, behavioral and blood pressure characteristics, by comorbidity, N = 2761.

	Total	No Co-Morbidity	Co-Morbidity	*p*-Value
	(N = 2761)	(N = 2521)	(N = 240)	
Demographic characteristics				
**Age (years)**				<0.001
Median (Q1, Q3)	56.0 (40.0, 66.0)	55.0 (39.0, 65.0)	64.0 (56.0, 71.0)	
**Sex**				<0.001
Male	915 (33.1)	866 (34.4)	49 (20.4)	
Female	1846 (66.9)	1655 (65.6)	191 (79.6)	
**Currently working**				<0.001
Yes	532 (34.5)	509 (36.4)	23 (16.4)	
No	1008 (65.5)	891 (63.6)	117 (83.6)	
**Residence**				0.611
Urban	1881 (68.1)	1721 (68.3)	160 (66.7)	
Rural	880 (31.9)	800 (31.7)	80 (33.3)	
Socioeconomic characteristics				
**Years of schooling**				<0.001
Median (Q1, Q3)	10.0 (7.0, 12.0)	10.0 (7.0, 12.0)	8.0 (6.0, 10.0)	
**Completed high school?**				0.003
Yes	652 (29.5)	615 (30.3)	37 (19.9)	
No	1561 (70.5)	1412 (69.7)	149 (80.1)	
**Household wealth tertile**				0.008
1 [lowest]	751 (33.4)	665 (32.6)	86 (41.3)	
2	749 (33.3)	678 (33.2)	71 (34.1)	
3 [highest]	748 (33.3)	697 (34.2)	51 (24.5)	
**Support from government?**				<0.001
Yes	936 (34.7)	827 (33.5)	109 (47.6)	
No	1764 (65.3)	1644 (66.5)	120 (52.4)	
Anthropometric characteristics				
**Waist-to-height ratio**				<0.001
Median (Q1, Q3)	0.6 (0.5, 0.7)	0.6 (0.5, 0.7)	0.6 (0.6, 0.7)	
**Body mass index (BMI) category, kg/m^2^**				0.012
Underweight (<18.5)	60 (3.2)	57 (3.3)	3 (1.9)	
Normal weight (18.5–24.9)	494 (26.1)	467 (27.0)	27 (16.7)	
Overweight (25–29.9)	538 (28.5)	490 (28.3)	48 (29.6)	
Obese (≥30)	799 (42.3)	715 (41.4)	84 (51.9)	
**Normotensive (<120/80 mmHg)**				0.065
Yes	1342 (48.6)	1239 (49.1)	103 (42.9)	
No	1419 (51.4)	1282 (50.9)	137 (57.1)	
**Pre-hypertensive (120/80-139/89mmHg)**				0.466
Yes	1385 (50.2)	1270 (50.4)	115 (47.9)	
No	1376 (49.8)	1251 (49.6)	125 (52.1)	
**Hypertensive**				<0.001
Yes	1168 (42.3)	1041 (41.3)	127 (52.9)	
No	1593 (57.7)	1480 (58.7)	113 (47.1)	
Behavioral characteristics				
**Add salt at table**				<0.001
Yes	1910 (69.4)	1770 (70.4)	140 (58.8)	
No	842 (30.6)	744 (29.6)	98 (41.2)	
**Self-reported vigorous intensity physical activity**				0.468
Yes	366 (13.3)	338 (13.5)	28 (11.8)	
No	2376 (86.7)	2167 (86.5)	209 (88.2)	
**Ever used alcohol?**				0.221
Yes	523 (19.0)	485 (19.3)	38 (16.0)	
No	2227 (81.0)	2028 (80.7)	199 (84.0)	
**Ever used tobacco?**				0.781
Yes	482 (17.5)	442 (17.6)	40 (16.9)	
No	2267 (82.5)	2070 (82.4)	197 (83.1)	

Frequencies and percentages in parenthesis are shown for categorical data. *p*-values shown are for Mann–Whitney U test for continuous data (normality test checked using the Shapiro–Wilk test); and chi-square test for categorical data, and Fisher’s exact test for BMI.

**Table 3:** Results from multivariate logistic regression model for hypertension and diabetes by demographic, socioeconomic, anthropometric, and behavioral factors.

VARIABLE	Description	HYPERTENSION	DIABETES
		aOR (Credible Interval)	aOR (Credible Interval)
Demographic characteristics			
**Age (years)**		1.06 (1.05; 1.08)	1.05 (1.03; 1.07)
**Sex**			
	Male	Reference	Reference
	Female	2.45 (1.62; 3.71)	2.36 (1.41; 4.01)
Socio-economic status characteristics			
**Household wealth tertile**			
	1 [lowest]	Reference	Reference
	2	0.90 (0.60; 1.34)	0.59 (0.36; 0.97)
	3 [highest]	0.83 (0.51; 1.35)	0.53 (0.28; 0.98)
**Support from government?**			
	No	Reference	
	Yes	3.77 (0; 1.6 × 10^8^)	
**Years of schooling**		0.90 (0.85; 0.95)	0.93 (0.87; 0.99)
Anthropological characteristics			
**BMI**		1.03 (1.01; 1.05)	
Behavioral characteristics			
**Add salt at table?**	No		
	Yes	1.48 (0; 2.9 × 10^8^)	
**Ever used tobacco?**	No		Reference
	Yes		1.74 (1.04; 2.88)

aOR—adjusted odds ratio.

## Supplementary Materials

The following are available online at https://www.mdpi.com/1660-4601/18/1/359/s1, Table S1: Population size and sample size, by province, Table S2: Results of fitting stepwise backward elimination multiple logistic regression models for factors associated with hypertension and diabetes (*p* = 0.10).
