# Supplementary material for: Bivariate Joint Spatial Modeling to Identify Shared Risk Patterns of Hypertension and Diabetes in South Africa: Evidence from WHO SAGE South Africa Wave 2
Source: Int J Environ Res Public Health. 2021 Jan 5;18(1):359. doi: 10.3390/ijerph18010359 (PMC7796507; doi:10.3390/ijerph18010359)
Supplement: Supplementary file 1 [file ijerph-18-00359-s001.pdf]

Supplementary Table 1: Population size and sample size, by province.

Province	Population estimate	% of total population	Number of 50+ sampled	% of 50+ sampled
	N=54,956,900		*N=1,817*	
Eastern Cape	6,916,200	*12.6*	287	*15.8*
Free State	2,817,900	*5.1*	157	*8.6*
Gauteng	13,200,300	*24.0*	321	*17.7*
KwaZulu-Natal	10,919,100	*19.9*	311	*17.1*
Limpopo	5,726,800	*10.4*	108	*5.9*
Mpumalanga	4,283,900	*7.8*	220	*12.1*
Northern Cape	1,185600	*2.2*	62	*3.4*
North West	3,707,000	*6.7*	116	*6.4*
Western Cape	6,200,100	*11.3*	235	*12.9*

Source: Statistics SA 'Mid-year population estimates' (2015) Table 2 p 2.

Supplementary Table 2: Results of fitting stepwise backward elimination multiple logistic regression models for factors associated with hypertension and diabetes (p=0.10).

VARIABLE	Description	HYPERTENSION		DIABETES	
		**aOR (95% CI)**	**P-value**	**aOR (95% CI)**	**P-value**
Demographic characteristics					
**Age (years)**		1.05 (1.04; 1.06)	<0.001	1.04 (1.03; 1.05)	<0.001
**Sex**					
	Male	Reference		Reference	
	Female	2.08 (1.46; 2.98)	<0.001	1.86 (1.3; 2.65)	0.001
Socio-economic status characteristics					
**Household wealth tertile**					
	1 [lowest]	Reference		Reference	
	2	0.82 (0.57; 1.16)	0.257	0.59 (0.42; 0.85)	0.004
	3 [highest]	0.63 (0.41; 0.97)	0.037	0.42 (0.27; 0.64)	<0.001
**Support from government?**					
	No	Reference			
	Yes	1.37 (1.00; 1.87)	0.046		
**Years of schooling**		0.91 (0.87; 0.96)	<0.001	0.93 (0.88; 0.97)	0.002
Anthropological characteristics					
**BMI**		1.03 (1.01; 1.05)	0.004		
Behavioral characteristics					
**Add salt at table?**	*No*				
	Yes	1.85 (1.37; 2.50)	<0.001		
**Ever used tobacco?**	*No*			Reference	
	Yes			1.62 (1.12; 2.32)	0.010

CI- Confidence Interval; aOR- adjusted odds ratio; Results are from multivariate logistic regression models adjusting for all the potential covariates listed in Table 2. Only significant variables are reported
